# Impact of different antithrombotics on the microcirculation and viability of perforator-based ischaemic skin flaps in a small animal model

**DOI:** 10.1038/srep35833

**Published:** 2016-10-21

**Authors:** Andreas M. Fichter, Lucas M. Ritschl, Luisa K. Robitzky, Stefan Wagenpfeil, David A. Mitchell, Klaus-Dietrich Wolff, Thomas Mücke

**Affiliations:** 1 Department of Oral and Cranio-Maxillofacial Surgery, Technische Universität München, Klinikum Rechts der Isar, Munich, Germany; 2 Institute for Medical Biometry, Epidemiology and Medical Informatics, University of Saarland, Homburg Saar, Germany

## Abstract

The effects of antithrombotic drugs on random and free flap survival have been investigated in the past, but the experimental and clinical results are not in agreement. A perforator-based critical ischaemia model was used to evaluate the effects of different perioperatively administered pharmaceutical agents on tissue ischaemia and to assess the potential additional haemorheological or vasodilative effects of antithrombotics on flap microcirculation. Combined laser Doppler flowmetry and remission spectroscopy revealed an increase in certain microcirculation parameters in most groups in comparison with saline controls, and these changes correlated with flap survival. Clopidogrel and hirudin significantly improved the amount of viable flap tissue in comparison with controls, while unfractioned heparin had a negative effect on flap survival. Low molecular weight heparin, aspirin, pentoxifylline, and hydroxyethyl starch had no impact on the amount of viable flap tissue. A higher complication rate was observed in all experimental groups, but only clopidogrel had a negative impact on the flap viability. Our results add to the body of evidence supporting the conclusion that perioperative antithrombotic treatment improves flap survival. Clopidogrel and hirudin are effective pharmacological agents that significantly increased the viability of perforator-based skin flaps in rats, but at a higher risk of postoperative bleeding.

Flap necrosis is an important complication in the field of plastic and reconstructive surgery. The key reasons for flap necrosis are usually attributed to intrinsic haemodynamic problems associated with the flap, including arterial flow insufficiency, venous congestion, or a combination thereof. Regardless of the cause, flap necrosis may require multiple revision surgeries with all of the associated risks, and usually results in prolonged wound care and hospitalization, work loss, unsatisfactory results, a reduced quality of life and increased medical costs^1^,^2^.

Numerous strategies have been proposed to avoid flap ischaemia, including surgical technique refinement, patient selection and perioperative planning^3^, tissue warming^2^ and the use of numerous pharmacological agents, such as vasodilators, anti-inflammatory drugs, sympatholytics, haemorheological agents and antithrombotics^4^,^5^,^6^,^7^,^8^,^9^,^10^,^11^,^12^,^13^,^14^,^15^. Encouraged by a body of evidence from animal experiments showing a positive impact of antithrombotic drugs on the patency rate of anastomoses in microvascular free flaps^4^,^5^,^6^,^7^,^8^,^9^,^10^,^11^,^12^,^13^,^14^,^15^ and on the survival of random^1^,^4^,^5^,^16^,^17^ and pedicle flaps^18^, many reconstructive surgeons today rely on peri- and postoperative antithrombotics^19^,^20^,^21^,^22^,^23^. However, the clinical usefulness of this practice has been debated in the recent past^24^. Antithrombotic treatment also has a higher risk of postoperative bleeding, an important complication that is rarely taken into account in animal studies investigating the effect of antithrombotics on flap viability. In most animal models used to study this subject, a thrombogenic environment is induced, e.g., using venous “tuck” or “crush-avulsion-injury” models^25^,^26^. However, these thrombogenic conditions do not correspond well with the clinical situation, which may explain the discrepancy of the effectiveness of antithrombotic drugs observed between animal experiments and the clinical setting^24^.

The aim of this study was to investigate the effects of different antithrombotic agents on flap ischaemia. To better reflect the clinical situation, a critical ischaemia model^27^ was used instead of creating a thrombogenic environment. Postoperative complications were documented and scored according to their incidence and severity.

Apart from the well-studied antithrombotic effects, additional anti-inflammatory^4^, vasodilative^28^,^29^ and/or haemorheological effects^5^ have been assumed for numerous antithrombotic agents such as acetylsalicylic acid, heparin and clopidogrel. Instead of limiting the focus of our study to the vessel patency^6^,^7^,^9^,^10^,^11^,^14^,^30^,^31^,^32^,^33^,^34^, another advantage of the surgical model used in this study was that it was possible to monitor the microcirculation changes in the skin and assess the actual clinical outcome (flap vitality), thereby further assessing possible additional drug effects.

## Results

All 108 recipient rats survived the postoperative period and tolerated the anaesthesia and operation well.

### Microcirculation changes

**Figure 1:**
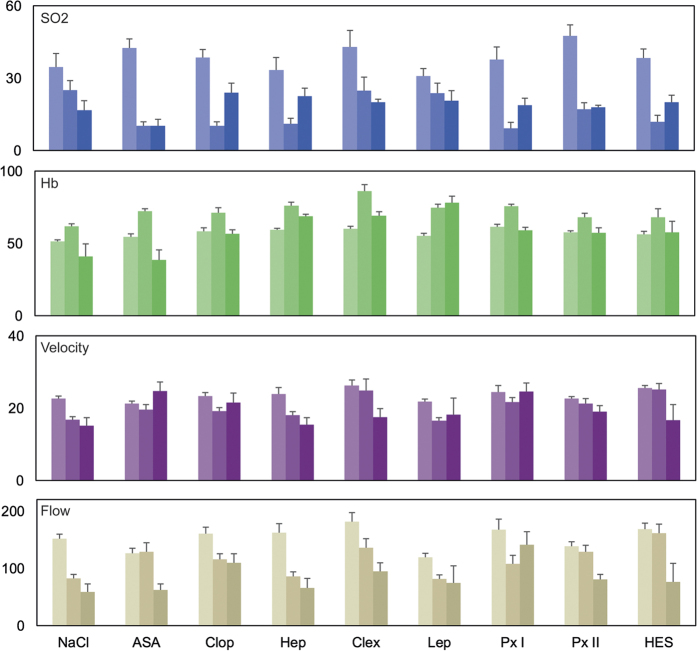
Impact of various pharmaceutical agents on the microcirculation. Depicted are the mean laser spectrophotometric values for the oxygen saturation (SO_2_ in percent), haemoglobin levels (Hb in AU), blood velocity (in AU) and blood flow (in AU) over the course of the experiment. Measurements were conducted preoperatively (light), after raising the flap (medium) and on day 7 (dark). Error bars indicate the standard error. *Abbreviations*: (NaCl) saline solution, (ASA) acetylsalicylic acid, (Clop) clopidogrel, (Hep) heparin, (Clex) clexane, (Lep) lepirudin, (Px) pentoxifylline, and (HES) hydroxyethyl starch.

The changes in the microcirculation system are depicted in Fig. 1. After raising the flap, a significant decrease in the blood flow, velocity and oxygen saturation and an increase in the haemoglobin level could be observed in all groups in comparison with the baseline values. The blood flow parameters (flow and velocity) tended to be higher in most experimental groups in comparison with the saline controls after the flap was raised. Hydroxyethyl starch (HES) and acetylsalicylic acid (ASA) seemed to have a positive influence on the blood flow in the microcirculation system. After seven days, however, this effect was less evident. The microcirculation parameters tended to recover in most groups with the exception of the groups administered saline solution, unfractioned heparin, low molecular weight heparin (clexane) and HES. The highest flow and velocity values were observed after the administration of clopidogrel or pentoxifylline (Px I). With the exception of saline solution and lepirudin, a hirudin derivative, the haemoglobin concentration returned to normal by day 7. The oxygen saturation tended to recover in the groups treated with clopidogrel, unfractioned heparin, pentoxifylline and HES, but did not reach the base values in any of the groups. Due to high interindividual fluctuations due to the method used^35^, no statistical comparisons between groups were performed.

### Flap viability on day 7 and postoperative complications

**Figure 2:**
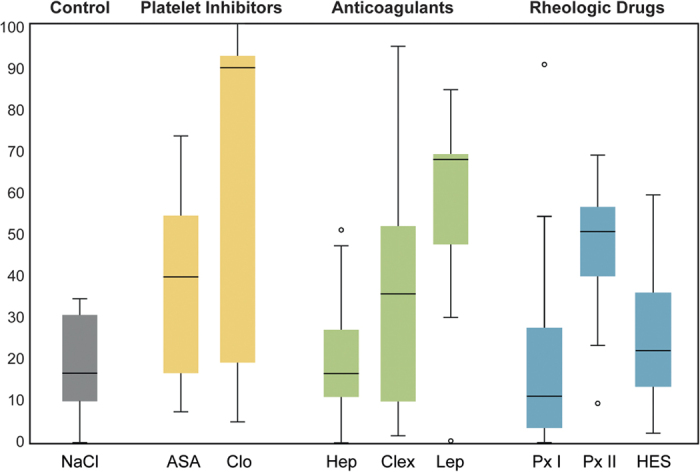
Impact of various pharmaceutical agents on the flap vitality. Box plot graph illustrating the amount of vital tissue (in percent) in the control group (saline solution, NaCl) and the experimental groups ((ASA) acetylsalicylic acid, (Clop) clopidogrel, (Hep) heparin, (Clex) clexane, (Lep) lepirudin, (Px) pentoxifylline, (HES) hydroxyethyl starch). The small circles indicate statistical outliers. Clopidogrel and lepirudin showed statistically significantly larger vital flap areas in comparison with saline controls.

The mean vitality rates are depicted in Fig. 2. The clopidogrel (median = 89.67, mean = 63.53 ± 38.16, *P* = 0.0003) and lepirudin (median = 67.50, mean = 58.03 ± 23.10, *P* = 0.003) groups showed statistically significantly larger vital flap areas in comparison with saline controls (median = 16.62, mean = 18.87 ± 10.95, Table 1). ASA, clexane and pentoxifylline (the latter only if administered 14 days prior to surgery) also showed a tendency to improve the flap survival in comparison with saline controls, but the differences were not statistically significant.

In the multivariate analysis, clopidogrel (*P* = 0.002) and lepirudin (*P* = 0.019) had a statistically significant positive influence on flap survival, while heparin (*P* = 0.03) showed a negative influence on flap survival. Pentoxifylline I (*P* = 0.054) also tended to have a negative influence on flap survival, but this effect failed to reach statistical significance. The other groups showed no statistically significant impact on flap survival.

The incidence and severity of postoperative complications are depicted in Table 2. The development of necrosis was significantly correlated with all microcirculation parameters on day 7, but not the parameters measured immediately after raising the flap (Table 3). The risk of haematoma was increased in all experimental groups, with an incidence rate between 67% and 100%. The most frequent and severe cases were observed in the clopidogrel, clexane, lepirudin and HES groups (Table 2). The microcirculation parameters, however, were not affected by haematoma formation (Table 3). However, individual examination of each group indicated that the haematoma severity did have a statistically significant (negative) impact on flap viability, although only in the clopidogrel group (*P* = 0.031, *r* = −0.621). Notable seromas were only observed if either HES or saline solution was administered (Table 2).

## Discussion

A number of antithrombotic drugs have shown a positive effect on the free flap patency rate^4^,^5^,^6^,^7^,^8^,^9^,^10^,^11^,^12^,^13^,^14^,^15^ and viability of random^1^,^4^,^5^,^16^,^17^ and pedicle flaps^18^ in animal experiments. However, clinical studies often fail to confirm a statistically significant advantage of postoperative antithrombotic treatment^24^, and the clinical usefulness of this practice has therefore been debated in the recent past^24^. One important reason for the discrepancy between animal models and clinical studies may be that almost all animal studies have been conducted under a thrombogenic environment^24^. Since these conditions do not closely resemble the clinical situation, it is likely that, although the influence of antithrombotics may not be significant in routine cases with low thrombogenic risks, their effect may be greater under thrombogenic conditions^24^. Differences observed between the outcomes of clinical and animal models may originate in the inadequacy of experimental models. To overcome this obstacle, we used a critical ischaemia model. The focus of our study was to assess the impact of different pharmaceutical agents on tissue ischaemia without creating thrombogenic conditions. A perforator flap model was chosen because it best resembles the anatomical aspects of a microvascular free flap, although (admittedly) without anastomosis (see limitations). At the same time, our model represents a critical ischaemia model comparable with a random flap model^1^,^4^,^5^,^16^,^17^, in which the amount of viable tissue correlates with the “anti-ischaemic” potential of the administered pharmaceutical agent^27^. The primary outcome parameter in our study was the amount of vital flap tissue seven days post-surgery. We feel that this approach to a small animal model more closely resembles clinical reality. Moreover, the complication rate and possible additional haemorheological effects apart from an antithrombotic effect could also be accounted for in our study. Only male animals were used to avoid a potential bias arising from fluctuations in the oestrus cycle, which can affect drug pharmacodynamics and pharmacokinetics^36^, coagulation^37^ and haemodynamics^38^ and can even have an impact on postoperative bleeding complications^39^,^40^.

Our study supports the assumption that many antithrombotic drugs also have an additional rheological effect, as other authors have proposed^14^,^15^,^41^,^42^,^43^,^44^,^45^. As could be expected from the previous studies^27^,^46^,^47^, the anatomical changes caused by raising a flap led to a temporary impairment in the microcirculation in all groups, with decreasing flow parameters and oxygen saturation. At the same time, rising haemoglobin levels were observed, indicating transient venous congestion. HES and ASA seemed to have had a positive influence on the blood flow in the microcirculation system directly after raising the flap, but this effect could not be observed after seven days. In comparison with saline controls, clopidogrel, clexane and pentoxifylline seemed to have had a positive influence on all microcirculation parameters on day 7. There was a strong negative correlation between the rate of necrosis and all microcirculation parameters on day 7. This finding indicates that in flaps with a high amount of necrotic tissue, the microcirculation in the (clinically) vital parts of the flap was also impaired. Further details on the influence of each pharmaceutical agent are discussed later in this paper.

*Acetylsalicylic acid* (ASA), or Aspirin^®^, irreversibly and non-selectively inhibits the COX-1 receptor, reducing the amount of thromboxane production that is essential for platelet aggregation. Apart from its common use in the treatment of coronary heart disease, ASA is also used in reconstructive microsurgery^48^. Shalom *et al.* noted that the well-known anti-inflammatory effects of ASA might be partly responsible for its positive effects on the survival of random skin flaps in rats^4^. Only ASA dosages (200 mg/kg body weight) had a positive impact on flap survival. In our study, an ASA dose of 25 mg/kg body weight led to increased vitality rates (38.1%) in comparison with saline controls (18.9%), but the difference failed to reach statistical significance. The dose used by Shalom *et al.* was approximately eight times higher than that used in our study, which would dramatically increase the potential risk of gastric ulcers or internal bleeding in the clinical setting. ASA had a positive initial impact on the microcirculation after the flap was raised, leading to higher flow and velocity values, but this effect was no longer evident after seven days.

*Clopidogrel*, another commonly used platelet inhibitor, specifically inhibits the P2Y_12_ subtype of the ADP-receptor, which is important for platelet activation and the cross-linking of fibrin. Clopidogrel is a prodrug that is converted to its active metabolite after being metabolized by hepatic cytochrome enzyme P2C19. Due to variable oral absorption, the bioavailability shows high interindividual variability^1^. Clopidogrel led to significantly higher patency rates in venous^6^ and arterial^7^ “tuck” models and significantly prolonged the time until a thrombus was formed in comparison with saline controls^6^. In rat random skin flap models, clopidogrel produced a significant increase in the survival rates^1^,^28^,^29^. Our perforator-based flap model corroborates these findings: with 63.5% viable tissue (median = 89.7%), clopidogrel was associated with the highest flap survival in our study. The vitality rate was significantly higher in the clopidogrel group in comparison to the saline solution, pentoxifylline and HES groups.

While the prevention of platelet aggregation is the most probable cause of the improvement noted in the viability of flaps, vasodilation, prevention of free O_2_ radicals and an effect on ischaemic reperfusion have also been discussed as possible mechanisms^28^,^29^. In fact, the microcirculation parameters were superior in the clopidogrel group in comparison with saline controls, indicating that an additional effect, due to vasodilatation or improved haemorheology, may have also played a role. In the logistic regression analysis, clopidogrel also had the highest impact on flap perfusion. However, with an incidence of 92%, haematoma formation was a problem in this study group. As the high median indicates, most flaps showed a very high amount of viable tissue, while few flaps became almost completely necrotic. These necrotic flaps all showed severe haematomas. The correlation analysis substantiates the suspicion that haematoma formation may have been responsible for flap necrosis in these cases. Based on the current literature and the results from this study, clopidogrel is likely to have a positive impact on flap survival. However, postoperative bleeding must be taken into consideration as a complication that can impede flap success.

*Heparin*, the glycosaminoglycan is the most commonly used anticoagulant^49^. Heparin also plays a role as an irrigant in microvascular surgery both in the unfractioned and low molecular weight forms^50^. While unfractioned heparin reduces the formation of fibrin clots by simultaneous inhibition of antithrombin III, thrombin and factor X, low-molecular-weight heparin (such as clexane) has a higher selectivity for factor X^12^. The vasodilative effect of heparin has also been discussed^51^. In experimental “crush-avulsion-injury” models, heparin increases the patency rate of anastomosed vessels^9^,^10^,^11^, and in high doses, even topical administration seems to effectively reduce systemic effects and associated risks^11^. Statements regarding the effects of heparin on flap viability are diverse and contradictory in the literature. While some authors have observed a significant improvement of the flap viability in random skin flaps^17^,^52^ or venous congestion models^53^, other authors have not^1^,^4^. In our experimental model, heparin failed to show a significant impact on flap viability. Low molecular weight heparin (36.7%) tended to show better results than unfractioned heparin (21.2%), which had the least positive effect on flap vitality of all experimental groups. The better results obtained using clexane may be attributable to its superior dose-response relationship^51^. Due to its pharmacodynamics, unfractioned heparin is usually administered as a continuous infusion clinically, which was not feasible in our animal model and may detract from the transferability of results. A positive effect on the microcirculation was not evident after heparin administration. Blood loss during surgery activates the coagulation system and induces hypercoagulability. Therefore, intraoperative heparin administration is considered to be protective. However, the initial administration of heparin is usually postponed until after the surgery is completed for fear of excessive intraoperative bleeding^51^. Systemic administration of heparin has often been associated with a higher risk of wound haematomas, which might impair flap perfusion and even lead to flap failure^51^,^54^. In this study, almost all animals treated with either unfractioned or low-molecular weight heparin (clexane) showed varying degrees of haematomas, but this process had no influence on flap viability.

*Hirudin*, a fibrinolytic substance extracted from the saliva of leeches, is the most potent natural inhibitor of thrombin. It works without antithrombin III and even inhibits clot-bound thrombin^55^. Desirudin, a synthetic hirudin derivative, significantly increased the patency rate of rat femoral veins in comparison with saline solution after microvascular anastomosis using the “tuck”^25^ model^12^. In one animal, a postoperative thrombus spontaneously dissolved in the desirudin group. In contrast to low-dose hirudin (250 μg/ml), intraluminal injection of high-dose hirudin (750 μg/ml) significantly decreased the thrombosis rate of traumatised rabbit ear arteries in comparison with saline solution^11^. Our study corroborates the positive effect of hirudin on flap perfusion. In the present study, we used lepirudin (Reflundan^®^), another synthetic hirudin derivative. This substance was withdrawn from the market in 2012, but we used some remaining samples, accepting the limited transferability to clinical practice. With a vitality rate of 58%, the amount of vital tissue was comparable to that resulting from treatment with clopidogrel (63.5%) and was significantly higher compared to the groups treated with saline solution, pentoxifylline I and HES. Since the drug intake has to be commenced two to four weeks prior to the surgical procedure to be fully effective^43^, the positive effects of lepirudin might have been even higher if the therapy had been commenced earlier. Hirudin also seems to have a positive, load-reducing effect on the venous system and improves microcirculation^56^. In accordance with the findings of previous studies, in our experimental model, the flow and oxygen levels in the microcirculation tended to be higher in the lepirudin group in comparison with saline controls, but no statistical analysis was performed.

The xanthin derivative *pentoxifylline* is a competitive nonselective photodiesterase inhibitor that reduces inflammation and innate immunity^57^. In addition, pentoxifylline increases the blood flow by inducing vasodilatation, inhibits platelet aggregation, reduces the concentration of fibrin and increases the deformability of erythrocytes (known as a haemorheological effect)^45^,^51^. A positive influence on the microcirculation and oxygenation of free flaps has also been reported^5^,^13^,^58^. Murthy *et al.*^13^ observed significantly higher vitality rates of rat epigastric flaps and higher patency rates of the supplying femoral/epigastric arteries after daily administration of pentoxifylline or low molecular weight heparin, while a combination of the two drugs had no significant impact on flap survival. In their study, a thrombogenic environment was created by traumatizing the vessel wall with a vascular clip. The fact that the combination therapy did not yield better results than controls might be attributable to haematoma formation under the skin island caused by the double anticoagulation^13^. The timing of the initial pentoxifylline dose seems to have an impact as well. For example, when the drug was administered every 12 hours in all study groups in a rat random skin model^5^, pentoxifylline only had a significant impact on flap survival if the first dose was administered directly before surgery. In contrast to this observation, the initiation of pentoxifylline therapy 14 days prior to surgery tended to have better results than administration directly prior to surgery in our study, but the difference was not statistically significant. Confirming the results of other studies that have indicated a positive effect on microcirculation^5^,^13^,^58^, pentoxifylline showed the highest flow and velocity values on day 7, but only when the initial dose was administered directly prior to surgery. However, the vitality rate was among the lowest of all experimental groups and did not differ from that of the saline controls. One reason for this poor result may have been that the pentoxifylline dose used in our study was too low to have an effect on flap viability. The dose used in our study was comparable to the human dose recommendation calculated down to match the weight of the animal, whereas the daily pentoxifylline dose used in the aforementioned studies was approximately four to five times higher.

*Hydroxyethyl starch* (HES), is a nonionic starch derivative. Due to its high water-binding capacity, which leads to a fluid shift from the interstitium to the intra-vascular space, HES is considered to be a plasma expander. In accordance with the Hagen-Poiseuille equation, HES increases the blood flow by causing haemodilution and a reduction of the blood viscosity. A direct platelet-inhibiting effect of HES has also been reported^59^. In a “crush-avulsion-injury” model, the patency rate of rabbit central ear arteries was significantly higher after the administration of dextran, another plasma expander^14^. However, no significant difference could be observed when the central ear arteries were conventionally severed and anastomosed. In a similar experiment, dextrane had only a temporary effect on the vessel patency during the first two hours after anastomosis. No long-term effect was observed^15^. Since HES was used instead of dextrane in our study, and the primary outcome was flap viability, a direct comparison with the aforementioned studies is not possible. Similar to dextran, however, HES showed a high blood flow (comparable with the baseline values) immediately after surgery, whereas the postoperative blood flow had dropped significantly in most of the other study groups. However, no positive long-term effect was observed, comparable with the aforementioned dextrane studies. HES had no positive impact on flap viability in our model.

All types of antithrombotics show trends towards increased risks of haematoma, regardless of the regimen. Heparin and ASA seem to increase this risk significantly in the clinical setting, as shown by a recent meta-analysis^24^. The authors argue that, given that haematomas can cause pedicle thrombosis and flap congestion, a correlation between flap haematoma and flap failure can affect the results^24^. Our results corroborate these findings. The risk of haematoma was significantly higher in all experimental groups in comparison with saline controls. The most severe cases of haematoma were detected in the clopidogrel, lepirudin and clexane groups, and haematoma formation had a significant negative impact on flap survival in the clopidogrel group. Wound haematomas were also observed in the control group but less often, and they were far less severe. Interestingly, microcirculation appeared to be unaffected by haematoma formation.

Flap losses after microvascular free flap transfer occur in 3.8% of cases^60^, and another 7.5%^60^ of flaps develop partial necrosis that can lead to wound infections, revision surgeries, prolonged hospital stays, inferior aesthetic and functional results and high economic costs. Our results add to the body of evidence indicating that the use of antithrombotic agents, especially the use of clopidogrel, has a positive effect on skin and tissue perfusion, which could be beneficial in the treatment of critically perfused flaps. Our results also impressively demonstrated the other side of the coin: there was an increased incidence of postoperative bleeding and haematoma formation, especially after clopidogrel treatment. Unfortunately, the most potent drug in our study also seems to be the most dangerous one with regard to postoperative complications. In light of these observations, the clinical use of antithrombotics to improve flap viability must be critically challenged, and the question needs to be raised as to whether the potential benefits of these pharmaceutical agents outweigh the higher risk of bleeding complications associated with their use.

Continued clopidogrel monotherapy among patients undergoing invasive procedures such as cardiac^61^ or spinal surgery^62^ can lead to potentially life-threatening complications. However, minor surgical interventions, such as oral surgery or local flap surgery under continued clopidogrel treatment, are either not associated with a high risk for postoperative complications^63^,^64^, or these complications can be managed with simple local measures^63^,^64^,^65^. Notably, postoperative dual platelet inhibition (clopidogrel and acetylsalicylic acid), as routinely performed 24 to 48 hours after cardiac surgery, does not cause any significant increase in bleeding risk when compared with acetylsalicylic acid monotherapy^66^.

With these findings in mind, we conclude that postoperative clopidogrel treatment is justified for minor interventions involving local flaps and may have a positive influence on flap perfusion based on our results. The raising of microvascular free flaps itself, however, can be considered an invasive procedure and is usually performed in conjunction with extended tumour resections. Therefore, the indications for postoperative clopidogrel treatment should be restricted to selected cases. As a recent case report^67^ shows, clopidogrel treatment, even if administered days after major surgery, can be beneficial in critically perfused flaps. Riml *et al.*^67^ salvaged a critically perfused rectus abdominis flap using a combination of clopidogrel and acetylsalicylic acid ten days after free flap transplantation and unsuccessful revision surgery. Although commencing clopidogrel treatment several days after major surgery reduces the postoperative risks, the full pharmacological potential of clopidogrel may be lost. With 3.8% flap losses and another 7.5% of flaps developing partial necrosis after free flap surgery^60^, it is all the more important to identify these cases early. Meticulous preoperative patient assessment can help to identify patients with a high risk of flap loss (e.g., severe atherosclerosis, coagulopathy, or a history of flap losses). Perioperative close monitoring of flap perfusion using established tools, such as indocyanine green angiography^68^ non-invasive laser Doppler flowmetry and remission spectrophotometry^69^ alongside clinical evaluations can help to identify critically perfused flaps early in the course of failure. In these high-risk cases, we believe that postoperative antithrombotic treatment is justified.

Our study demonstrates that various pharmacological agents positively influence skin perfusion and that some (clopidogrel and lepirudin) have the potential to significantly improve flap viability. However, a number of limitations should be taken into consideration when interpreting the results of this study. In general, experiments performed in rodents may not accurately predict the results in humans. The doses and frequencies used in this study were selected according to recommendations from the literature, or, if no comparable animal study existed, were specified according to human dose recommendations. Other doses or combinations of different pharmacological agents may lead to different results. Although flaps were raised as perforator-based “free” flaps in this study, no microvascular anastomosis was performed to avoid the creation of a thrombogenic environment. Therefore, a direct comparison of our model with microvascular *free* flaps might not be entirely adequate. Our model may be more adequately compared with a random skin flap model, in which the amount of tissue necrosis directly correlates with the effect of the administered pharmaceutical agent.

This study showed that clopidogrel and hirudin (lepirudin) are effective pharmaceutical agents that significantly increase the viability of perforator skin flaps in rats. Our study supports the assumption that many antithrombotic drugs also have an additional rheological effect. Our positive results are overshadowed by a significantly higher risk of postoperative bleeding complications after antithrombotic therapy, which had a significant impact on flap viability in the clopidogrel group. This study adds to the body of evidence supporting the conclusion that perioperative antithrombotic therapy can have a positive influence on flap survival, but further large-scale clinical studies are needed to confirm or disprove these observations and help to decide whether the marginal benefits in terms of flap perfusion and viability outweigh the higher risk of bleeding complications.

## Methods

All animals were treated and housed in accordance with EU guidelines. The study was approved by the regional government (Regierung von Oberbayern, AZ55.2-1-54-2532-129-10) and was conducted in accordance with the German Animal Welfare Act. A total of 108 male Wistar rats (280–320 g, Fa. Charles River, Kißlegg, Germany) were used. Food and water were provided *ad libitum*. All surgical procedures were performed under intravenous general anaesthesia [100 mg/kg ketamine (Narketan®, Fa. Vétoquinol GmbH, Ravensburg, Germany) and 5 mg/kg xylazine (Rompun®, Fa. Bayer Vital GmbH, Leverkusen, Germany)] and aseptic conditions as described elsewhere^70^, and all efforts were made to minimise suffering.

### Surgical technique

**Figure 3:**
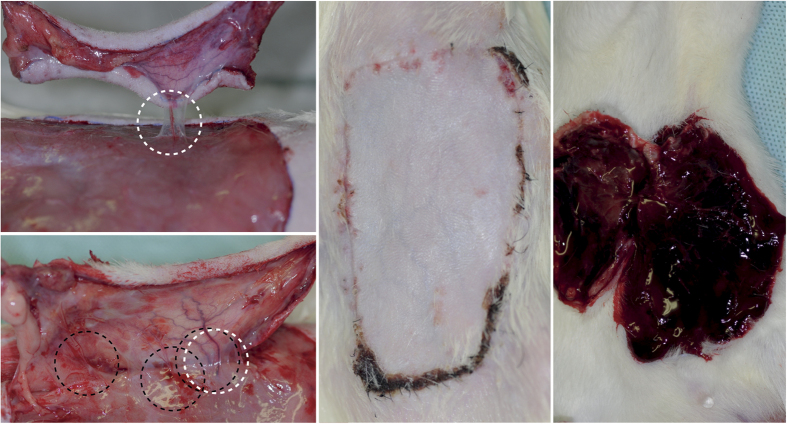
Clinical aspects of epigastric perforator flaps. *Upper left:* Raised epigastric flap based on a single perforator (white broken circle) after bipolar cautery of the superficial epigastric vessels and all but one deep inferior perforator. The remaining perforator was handled with great care to avoid vasospasm. *Lower left and middle:* The same flap on postoperative day 7 after daily clopidogrel treatment. *Lower left:* In addition to the strong, pulsating, main perforator (white circle), several new vessels (black circles) can be observed. *Middle:* The vital flap on day 7 after clopidogrel treatment with almost no necrotic area. The re-established vascular network is visible through the skin. *Right:* Massive haematoma under a necrotic flap on day 7 after clopidogrel treatment.

The epigastric flap was raised as a perforator flap based on a single main perforator from the deep inferior epigastric system with a standardised dimension of 4 × 7 cm^2^, as described elsewhere in detail (Fig. 3)^27^,^71^. An oversized flap model was selected to detect the possible impact of different pharmaceutical agents on the vitality of critically perfused areas of the flap^72^. After the flap was raised, it was sutured back into the wound bed with 6–0 monofilament interrupted sutures (Ethilon^®^, Ethicon, Norderstedt, Germany).

### Experimental groups

All rats were randomised by a computer-generated list and subdivided into nine groups (n = 12). Following a strict protocol, ASA, clopidogrel, unfractioned heparin, low molecular weight heparin (clexane), hirudin (lepirudin), pentoxifylline or HES was administered (n = 12 each) starting prior to surgery and continuing for seven days. Each group was treated perioperatively with either saline solution (control group) or an active medication according to the medication protocol depicted in Table 4. The doses and frequencies used in this study were selected according to recommendations from the literature, or, if no comparable animal study existed, were specified according to human dose recommendations.

### Assessment of microcirculation

In addition to observing the flap colour and capillary refill, measurements of the tissue oxygen saturation (SO2 in %), haemoglobin level (Hb, in AU, arbitrary units), blood flow (in AU) and velocity (in AU) were non-invasively performed using combined laser Doppler flowmetry and remission spectroscopy (O2C, equipped with an LF-2 probe, Lea Medizintechnik, Giessen, Germany). This technique is an established procedure used for the assessment of free flaps and has been described elsewhere in detail^69^. In all surgical groups, O2C was performed preoperatively (base value), after the flap was raised and on day 7. All measurements were taken from the central part of the flap and compared to the same area if possible. If necrosis had occurred, measurements were taken from the central area of the remaining viable flap tissue instead.

### Planimetric measurement of necrotic areas

On postoperative day 7, the rats were put under anaesthesia again, and flap healing was documented using a digital SLR camera (type Nikon Coolpix 8700. Nikon Corp., Chiyoda, Tokyo, Japan) mounted in a perpendicular direction to the flap with a tripod. Pictures were analysed with respect to vital and necrotic areas. Therefore, the total flap area and necrotic areas were manually circumscribed with the help of a graphic tablet, and the cross-sectional area was calculated using the ImageJ software program^73^.

### Assessment of postoperative complications

Rats were euthanised while still under deep anaesthesia using a combination of lethal pentobarbital injection (200 mg/kg, Narcoren^®^, Rhone-Merieux, Laupheim, Germany) and bleeding by severing the abdominal aorta. Flaps were raised as described above and all signs of haematoma or seroma were documented and scored according to the severity of the complication (“−” =none, “(+)” =very minor, “+” =minor, “++” =moderate, “+++” =severe).

### Statistics

For the basic statistical analysis, the Prism software (Prism 7 for Mac OS X, Version 7.0a, GraphPad Software, Inc., La Jolla, CA, USA) was used. The SPSS software package (SPSS 22, SPSS Inc., Chicago, IL, USA) was used for the multivariate analysis. The rate of necrosis was assessed using an ordinary one-way ANOVA to determine the significance of differences between groups. Tukey’s multiple comparison testing was used to account for the problem of multiple testing. Pearson’s correlation was used to determine whether postoperative haematoma formation had a significant influence on the flap viability. Differences were considered to be statistically significant for a two-sided *P* value of <0.05. All data are presented as the mean ± standard deviation (SD). All observations were independently evaluated by two investigators blinded to the experimental groups.

## Additional Information

**How to cite this article**: Fichter, A. M. *et al.* Impact of different antithrombotics on the microcirculation and viability of perforator-based ischaemic skin flaps in a small animal model. *Sci. Rep.* **6**, 35833; doi: 10.1038/srep35833 (2016).

## Figures and Tables

**Table 1:** Statistical analysis of the vitality rate.

	NaCl	ASA	Clop	Hep	Clex	Lep	Px I	Px II	HES
NaCl		0.539	**0.0003**	>0.999	0.637	**0.003**	>0.999	0.112	0.998
ASA			0.175	0.699	>0.999	0.489	0.761	0.995	0.933
Clop				0.0008	0.127	1.000	**0.001**	0.673	**0.005**
Hep					0.786	0.006	>0.999	0.191	>0.999
Clex						0.395	0.839	0.986	0.966
Lep							**0.009**	0.946	**0.028**
Px I								0.235	>0.999
Px II									0.457
HES									

Statistical analysis of the vitality rates between all groups. An ordinary one-way ANOVA was used for the statistical analysis. Tukey’s multiple comparisons test was used to account for the problems associated with multiple testing. The adjusted two-tailed *P* values are provided. *Abbreviations*: (NaCl) saline solution, (ASA) acetylsalicylic acid, (Clop) clopidogrel, (Hep) heparin, (Clex) clexane, (Lep) lepirudin, (Px) pentoxifylline, (HES) hydroxyethyl starch. Statistically significant differences (*P* < 0.05) are marked in bold.

**Table 2:** Postoperative complications.

	Control	Platelet Inhibitors		Anticoagulants			Rheologic Drugs		
	NaCl	ASA	Clop	Hep	Clex	Lep	Px I	Px II	HES
Necrosis	81.13 ± 10.95	61.90 ± 22.15	36.47 ± 14.93	78.84 ± 14.29	63.27 ± 29.26	41.97 ± 23.10	77.88 ± 26.38	53.80 ± 16.67	74.19 ± 16.58
Haematoma Incidence	4/12	8/12	11/12	11/12	12/12	12/12	11/12	10/12	12/12
Haematoma Severity	(+)	+	++	+	++	++	+	+	++
Seroma Incidence	9/12	7/12	6/12	0/12	0/12	1/12	6/12	1/12	9/12
Seroma Severity	+	(+)	(+)	−	−	−	(+)	−	+

The mean percentage of skin necrosis (±SD) and evidence of haematoma and/or seroma (Score: “−” = none, “(+)” = very small, “+” = small, “++” = moderate, “+++” = large) on postoperative day 7. *Abbreviations*: (NaCl) saline solution, (ASA) acetylsalicylic acid, (Clop) clopidogrel, (Hep) heparin, (Clex) clexane, (Lep) lepirudin, (Px) pentoxifylline, (HES) hydroxyethyl starch.

**Table 3:** Impact of postoperative complications on the microcirculation.

	SO_2_ day 0	Hb day 0	Flow day 0	Velocity day 0	SO_2_ day 7	Hb day 7	Flow day 7	Velocity day 7
Necrosis	−0.158 (0.102)	−0.177 (0.068)	0.026 (0.788)	0.012 (0.902)	**−0.326 (0.003)**	**−0.227 (0.038)**	**−0.437 (<0.0001)**	**−0.431 (<0.0001)**
Haematoma					0.031 (0.782)	0.086 (0.435)	−0.014 (0.896)	−0.100 (0.366)

Pearson’s correlation was used for the statistical analysis. The Pearson coefficient, *r*, and the two-tailed *P* values (in brackets) are provided. *Abbreviations*: (Day 0) the day of the operation after the flap was raised, (Day 7) postoperative day 7, (SO_2_) oxygen saturation, (Hb) haemoglobin level, (Flow) blood flow, (Velocity) blood flow velocity. The rates of both necrosis and haematoma were assessed on day 7. Not all comparisons made logical sense, and statistical tests were therefore not performed in these cases (empty boxes). Statistically significant differences (*P* < 0.05) are marked in bold.

**Table 4:** Surgical groups and medication protocol.

Time	Control	Platelet Inhibitors		Anticoagulants			Rheologic Drugs		
	NaCl	ASA	Clop	Hep	Clex	Lep	Px I	Px II	HES
	i.p.	i.p.	p.o.	s.c.	s.c.	s.c.	i.p.	i.p.	i.p.
−14 d								4.5	
0	7.5	25	4.5	100	100	0.25	4.5		7.5
Every 8 h				100		0.25			
Every 12 h			4.5		100				7.5
Every 24 h	7.5	25					4.5	4.5	

*Abbreviations*: (kgbw) kilograms of bodyweight, (IU) international units, (i.p.) intraperitoneal application, (p.o.) per orum, (s.c.) subcutaneous application, (d) day(s), (h) hour(s), (NaCl, in mg/kgbw) saline solution, (ASA, in mg/kgbw) acetylsalicylic acid, (Clop, in mg/kgbw) clopidogrel, (Hep, in IU/kgbw) heparin, (Clex, in IU/kgbw) clexane, (Lep, in mg/kgbw) lepirudin, (Px, in mg/kgbw) pentoxifylline, (HES, in ml/kgbw) hydroxyethyl starch.
